# Utilization of delivery care among rural women in china: does the health insurance make a difference? a cross-sectional study

**DOI:** 10.1186/1471-2458-10-695

**Published:** 2010-11-15

**Authors:** Shengbin Xiao, Hong Yan, Yuan Shen, Shaonong Dang, Elina Hemminki, Duolao Wang, Qian Long, Jianmin Gao

**Affiliations:** 1 Department of Epidemiology and Health Statistics, School of Public Health, Xi'an Jiaotong University College of Medicine, Xi'an, Shaanxi 710061, China; 2 THL, National Institute for Health and Welfare, P.O. Box 30, FI-00271 Helsinki, Finland; 3 Department of Medical Statistics, London School of Hygiene and Tropical Medicine, London WC1E 7HT, UK; 4 Department of Public Health, University of Helsinki, Finland; School of Public Health, Chongqing Medical University, China; 5 School of Public Policy and Management, Xi'an Jiaotong University, Xi'an, Shaanxi 710061, China

## Abstract

**Background:**

Since 2003, the New Cooperative Medical Scheme (NCMS) has been implemented throughout rural China, usually covering delivery services in its benefit package. The objective of this study was to compare the difference of utilization of delivery services, expenditures, and local women's perceived affordability between women with and without reimbursement from NCMS.

**Methods:**

A cross-sectional survey was carried out in two rural counties in Shaanxi province, China, during December 2008-March 2009. Women giving birth from April 2008 to March 2009 were interviewed by a structured questionnaire to collect information on utilization of delivery services. Multivariable analyses were used to compare the differences in outcomes between women with and without reimbursement from NCMS.

**Results:**

Of the total 1613 women interviewed, 747(46.3%) got reimbursement to cover their expenditure on delivery care (NCMS group) and 866(53.7%) paid delivery services entirely out of their own pocket (Non-NCMS group). Compared with the Non-NCMS group, the NCMS group had significantly more women who delivered at hospital. The rate of Caesarean section (CS), proportion of women seeking higher level services, and length of hospitalization were similar between the two groups. The total hospital costs for delivery services in the NCMS group was significantly smaller and after being reimbursed, the out-of-pocket payment in the NCMS group was less than a half of that in the Non-NCMS group. Fewer women in the NCMS group than in the Non-NCMS group considered their payment for delivery services expensive.

**Conclusions:**

There was no evidence of overuse delivery services among the women reimbursed by NCMS. Total hospital costs and women's costs for delivery services were found lower in the NCMS group, subsequently alleviation on women's perceived financial affordability.

## Background

It was estimated that over half a million women died each year during pregnancy, delivery or shortly thereafter, almost all of them occurred in developing countries [1,2]. It is widely acknowledged that most maternal mortality is avoidable, if an immediate and effective professional care were provided during and after labour and delivery [2], and hospital delivery has been promoted as in China [3]. While there are a number of socio-economic and cultural factors that act as barriers to women's use of health services, the high cost of services has been identified as a major barrier facing rural women in seeking and using these life-saving services in many developing countries including China [4-7]. Evidence from sub-Saharan Africa and South Asia showed that households often spent significant amounts for delivery care, especially if complications arise [8,9].

Before the economic reform in the late 1970 s, the healthcare for most Chinese rural residents was covered by the Cooperative Medical System (CMS), one type of community-based health insurance. The CMS was structured as a three-tiered healthcare delivery system - including village healthcare, township health centers, and county hospitals [10-12] - and played a vital role in improving the health of the rural population. However, the CMS collapsed in rural areas after the introduction of market reform in the late 1970 s and early 1980 s. Since the 1980 s, health care for the rural population shifted to a fee-for-service system which required rural people to pay most of their own healthcare [13]. According to the 1998 China National Health Service Survey, only 6.6% of villages had CMS, and more than 87% of rural residents did not have any health insurance [14]. Meanwhile, health care costs escalated and impoverished many rural residents. A number of local surveys showed that approximately 20-30% of all households in poverty were due to high medical expenses [15,16]. In response, one of the top priorities for the Chinese government since 2002 has been to re-establish the health care system in rural areas. Since 2003, a nationwide pilot project called the New Cooperative Medical Scheme (NCMS) has been implemented under the guidelines issued by the Chinese Central Government. The NCMS system aims to improve rural residents' access to health care services, reduce poverty due to illness, and provide financial risk protection to patients with serious illnesses [17,18]. The NCMS is a voluntary-based scheme and the content is decided at the county level [19].

The study areas involved two counties in Shaanxi province in northwest China, Zhen'an and Lantian, both typical of poor rural areas in China in terms of national GDP rankings. The local residents are mainly engaged in farming. There were 25 townships, comprising 204 villages in Zhen'an county, with a total population of about 290,000. And 29 townships, comprising 519 villages in Lantian county, with a total population of about 630,000. Zhen'an county started NCMS in 2003 and Lantian county in 2007. The delivery services have been covered into the benefit package of NCMS shortly after this new scheme being initiated in the two counties. Some studies[20-24] on NCMS for rural population have been carried out, but most of them focused on the capacity of NCMS in reducing financial risk and few studies could be found on the relationship between NCMS and health services utilization, and even fewer[25] on maternal healthcare utilization.

The objective of this study was to compare the difference of utilization of delivery services, expenditures, and local women's perceived affordability between women with and without reimbursement from NCMS.

## Methods

A cross-sectional survey was carried out in the two counties, during December 2008-March 2009. All townships in both counties and one third of their villages were selected from a list stratified by the population size and distance to the township hospital. The study population comprised resident women giving birth from April 2008 to January 2009 in Lantian and April 2008 to March 2009 in Zhen'an county, being identified by village doctors and township maternal healthcare workers. A structured questionnaire (Additional file 1) was used to collect information on demographic and socioeconomic background characteristics, birth history, maternity benefit package of the NCMS, utilization of maternal health care, expenditure and their perceived affordability during the last pregnancy. The questionnaire was pre-tested before the actual survey. Interviews were conducted at women's homes by trained interviewers who were researchers and postgraduate students from Xi'an Jiaotong University. If a woman was not at home at the time of the survey, her husband or some other family member responded on her behalf. The respondents' consent was asked orally. Interviews lasted about 20-30 minutes. Completed questionnaires were checked in the field for errors and omissions by the research team.

The survey obtained an ethical approval of the International Centre for Reproductive Health (ICRH) at the Ghent University, Belgium, and a local approval was obtained from the Ethics Committee of Xi'an Jiaotong University [26].

The interviewed women were divided into two groups: NCMS group and Non-NCMS group. Women who had got some reimbursement from NCMS or any other source for their delivery costs were allocated into NCMS group (n = 747), and women who paid all the costs themselves were allocated into Non-NCMS group (n = 866). In the NCMS group, the women only need to pay for the costs, which the NCMS does not cover, so the total hospital costs is the women's costs (the out-of-pocket payment) plus the reimbursement. In the Non-NCMS group, the total costs equals to the out-of-pocket payment in the number since there is no any reimbursement to cover their delivery.

Women receiving reimbursement from other sources were included in the NCMS group. This was done, as a few women maybe could not distinguish the sources of reimbursement. During the study period there were two projects reimbursing the facility-based delivery in Zhen'an county. One was a project to reduce maternal mortality and eliminate the newborn tetanus. It was financed by Chinese central government and targeted poor women with facility-based delivery. Another was a project to reimburse hospital delivery. It was financed by local government and targeted all women with facility-based delivery. The two projects may have covered all or part of the expense remaining after the NCMS reimbursement.

Statistical analyses were performed by using SPSS software. Descriptive analysis of variables was carried out on the basis of medians (or means) and corresponding percentages. Univariate analyses, including Mann-Whitney U-test and Chi-square test were used to examine the differences of women's background characteristics between the two groups. Earlier studies showed that women's age, parity, education level and her household income influence the utilization of maternity care [27,28], so multivariable analyses, including logistic regression, ordinal regression and linear regression were carried out to control for the confounding effects from these unbalanced characteristics of the women when comparing the outcome variables between the two groups. County was introduced into multivariable analyses as a categorical variable. A p-value of < 0.05 was considered statistically significant.

## Results

In total, 1614 women themselves (1071 in Lantian county and 543 in Zhen'an county) and 70 relatives answered the questionnaire (response rate of 76%). The reasons of non-response are as follows: no-one at home on the day of the survey (240, 10.8%), working out of their hometown (253, 11.4%), interviewers' transportation problems or other reasons (41, 1.8%); few (3, 0.1%) refused the interview. The data provided by relatives and one woman with lacking delivery data were excluded, leaving 1613 women for the analysis.

Out of 1613 women, 866(54%) paid all delivery costs out of their own pocket, and 747(46%) were reimbursed by health insurance. Among the women being reimbursed, 98% were reimbursed by NCMS, including 50(3%) who were also simultaneously reimbursed by other health insurance, and only 16(2%) were reimbursed solely by other health insurance.

There were more young and primiparous women and women with higher household income in the NCMS group than in the Non-NCMS group. The difference in parity was very large. No significant difference was found in regard to women's education level (Table 1).

**Table 1:** Demographic and socioeconomic characteristics of women in the two groups (number (%))

Characteristic	NCMS (n = 747)	Non-NCMS (n = 866)	Total	p*-*value
Age(years)				
< 20	2(0.3)	8(0.9)	10(0.6)	< 0.001
20-24	364(48.7)	282(32.6)	646 (40.0)	
25-29	241(32.3)	323(37.3)	564(35.0)	
30-34	115(15.4)	167(19.3)	282 (17.5)	
35+	25(3.3)	86(9.9)	111(6.9)	
Parity				
Primiparous	535 (71.6)	403(46.5)	938 (58.2)	< 0.001
Multiparous	212 (28.4)	463(53.5)	675 (41.8)	
Education				
Illiteracy	26(3.5)	35(4.0)	61 (3.8)	0.255
Primary school	129(17.3)	138(15.9)	267 (16.6)	
Junior high school	492(66.0)	558(64.4)	1050 (65.1)	
Senior high school or above	99 (13.3)	135 (15.6)	234(14.6)	
Household income(RMB, yuan)				
≤ 7000	83(11.9)	109(13.7)	192(12.9)	0.005
7001-10000	198(28.4)	257(32.4)	455(30.5)	
10001-20000	291(41.8)	309(39.0)	600(40.3)	
20001^+^	125(17.9)	118(14.9)	243(16.3)	

All women in the NCMS group and 96% in the Non-NCMS group had a hospital delivery. Among the women who gave birth at a hospital, the two groups were similar in regard to the hospital level and the mode of delivery. Most women gave birth at county or higher level hospital and had vaginal delivery. After adjusting for women's age, parity, maternal education, household income, distance to health facility and county, there were no statistically significant differences between the NCMS and Non-NCMS groups in the length of stay at hospital for delivery (Table 2).

**Table 2:** Use of delivery services in the NCMS and Non-NCMS groups (number (%))

	NCMS (n = 747)	Non-NCMS (n = 827)	Total
Place of delivery^#^			
Township level hospital	183(24.5)	201 (24.3)	384(24.4)
County level or above hospital	564 (75.5)	626(75.7)	1190(75.6)
p-value*	0.176		
Mode of delivery^#^			
Vaginal delivery	563(75.5)	639(77.3)	1202(76.4)
Caesarean section	183 (24.5)	188 (22.7)	371(23.6)
p-value*	0.053		
Days of hospitalization^#^			
0	31 (4.4)	26 (3.6)	57(4.0)
1-3	199 (28.2)	244 (33.5)	443(30.9)
4-6	211 (29.9)	197 (27.0)	408(28.4)
7-9	191 (27.1)	178 (24.4)	369(25.7)
10^+^	73 (10.4)	84 (11.5)	157(10.9)
p-value*	0.255		

* p-value was calculated from logistic regression model controlling for women's age, parity, education level, her household income, distance to health facility and county for the dependent variables "Place of delivery" and "Mode of delivery"; and calculated from ordinal regression model controlling for women's age, parity, education level, her household income, distance to health facility, county, place of delivery and mode of delivery for the dependent variable "Days of hospitalization".

^#^There were three independents, women's age, parity and household income, entering the model for the dependent variable "Place of delivery"; three independents, women's age, parity and county, entering the model for the dependent variable "Place of delivery"; and four independents, parity, county, place of delivery and mode of delivery entering the model for the dependent variable "Days of hospitalization".

The NCMS group had a smaller total payment for delivery than the Non-NCMS group, with a median difference of 300 RMB (44 USD, 1 USD = 6.8 RMB) and mean difference of 471 RMB (69 USD). The NCMS group had a reimbursement of on median 600 RMB (88USD) (mean 822 RMB, 121USD), which was a half of the total costs. The out-of-pocket payment was less than half in the NCMS group than in the Non-NCMS group (Table 3).

**Table 3:** Costs for facility-based delivery between the NCMS and Non-NCMS groups

	NCMS (n = 735)	Non-NCMS (n = 792)	Total
Total payment^#^			
mean ± SD	1920.04 ± 1897.57	2390.94 ± 2569.26	2164.28 ± 2282.33
Median	1200	1500	1250
p-value*	< 0.001		
Out-of-pocket payment^#^			
mean ± SD	1109.43 ± 1597.69	2403.44 ± 2592.02	1768.28 ± 2255.96
median	600	1500	1000
p-value*	< 0.001		

*p-value was calculated from linear regression model controlling for women's age, parity, education level, household income, place of delivery, mode of delivery, days of hospitalization and county. A logarithmic transformation for dependent variables was conducted when using linear regression model.

^#^Other than NCMS status, place of delivery, mode of delivery, days of hospitalization and county entered the two models of linear regression as independent variables.

**Figure 1:**
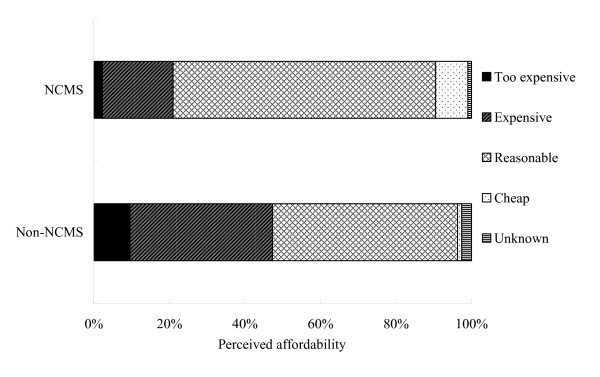
**Women's perceived affordability to out-of-pocket payment between the NCMS and Non-NCMS groups**.

Figure 1 compares the differences in women's perceived affordability of the out-of-pocket payment for facility-based delivery between the NCMS and Non-NCMS groups. Women in the Non-NCMS group more often considered their payment for delivery services to be expensive or too expensive, even after adjusting for women's background characteristics and county.

## Discussion

Obviously, it was rare for local rural women in study areas who had other health insurances than NCMS to cover their expenditures on delivery care. So generally the NCMS group here could almost make a full presentation of the NCMS member's characteristics.

In some previous studies in China [29,30], financial reimbursement from NCMS was identified as an important promote of hospital delivery. A study from Turkey [31] reported that health insurance had a positive impact on utilization of maternal health care. In our study counties, the rate of hospital delivery was high (98%) and further improvement was difficult. To obtain reimbursement from NCMS, delivery at hospital was a necessary condition explaining the 100% hospital delivery rate in the NCMS group. This regulation was established in NCMS to encourage hospital delivery.

The distribution of hospital's level was the same in the two groups, about a quarter of childbirth occurred at township hospital and others at county or higher level hospital in both groups. Our data indicated that the reimbursement from NCMS seems not to have influenced the choice of the hospital level. It is possible that this may be partly explained by a rule of NCMS that a relatively greater reimbursement was allotted to lower level hospitals. The women in the NCMS group may have weighed the bigger expenditure against the higher level services.

There was no indication of NCMS leading to overuse of health services, as measured by the length of hospital stay or cesarean section rate. Too long hospital stay may imply either an unnecessary medical waste or a health risk, including maternal or neonatal infections. No significant difference was found in the length of stay between the two groups.

Caesarean section is indicated in certain medical circumstances and those not at medical request could directly lead to increased risk of morbidity and mortality of the mother or newborn. In 1985, the World Health Organization proposed 15% as the highest acceptable rate of CS for all deliveries [32]. No significant difference in the rate of CS was found in our study between the NCMS and Non-NCMS groups, although the overall rate of CS remained higher than WHO recommendation.

Wagstaff and Lindelow (2008) analyzed data from three household surveys in China on the effect of health insurance on household financial risk and found that in all three surveys health insurance increased the risk of high and catastrophic spending and they suggested that it was due to different forms of supplier-induced demand[23]. This is an interesting finding that our data indicated that NCMS reimbursement did not result in higher delivery care expenditure, on the contrary, it directly generated a considerable reduction of the out-of-pocket payment and subsequently alleviation on women's perceived affordability. This is consistent with the findings of Wagstaff et al. (2009), who reported a reduction in expenditure for delivery services after the introduction of NCMS to cover the delivery services [24]. The possible reason for this result, we think, could be largely contributed to a different payment system. The fixed charges for episodes of care, particularly called charge for single disease, have been introduced in NCMS, in which delivery care was charged same as a single disease, instead of the system of fee-for-services, which is the most common mode of charging for medical services in China. This may have a restraining effect on the provider's incentive to provide unnecessary tests, drugs, hospitalization or other high-tech care, subsequently leading to a reduction of unnecessary costs. Of course, the enforced supervision in NCMS system on medical quality and performance of charging of hospital and doctors was perhaps another contributor.

The policy implication of our study is that the strategies of covering delivery care into NCMS benefit package have been successful in the study area. It might be appropriate to enlarge the coverage of NCMS to cover more maternal care, such as antenatal care and postnatal care. At the same time, it would be useful to continue enforcing the supervision on medical quality and charges to guarantee participators to get a genuine benefit from NCMS and to prevent unnecessary waste of scarce medical resources.

There are several limitations to be considered when interpreting the results of this study. Firstly, all the data used were collected retrospectively from women's report, and this may have yielded some recall bias. While this bias would not be too large since this study just targeted the women giving birth not more than one year before the survey. Secondly, actual reimbursement rather than NCMS membership was used as the basis of classification in this study. Some women with unauthorized pregnancies could not get reimbursement from NCMS even if they had participated in the NCMS. In addition, some insured women maybe did not claim the reimbursement for some reasons even they were entitled to. These women were all included in the Non-NCMS group and this may have introduced some selection bias if those women have some special characteristics. Although the results were adjusted by using the multivariable analyses to control the confounding effects of some socio-economic status and demographic characteristics, we still could not rule out the potential bias derived from some non-observable characteristics if they were not balanced between the NCMS and Non-NCMS groups. Thirdly, the rural population coming into city and becoming migrant workers is common in the study counties, as the same in other rural area in China. Some local women giving birth during the study period maybe have been left out if they didn't live in their hometown. These women were excluded from the target population. Also there were some women who lived in the study counties during their pregnancy, even used the antenatal care or (and) delivery care in local area and were identified as the target population by local healthcare personnel, but migrated into city shortly after childbirth. These women also might have been dropped out from the survey, and this case has become the main reason for non-response in this study. It is not clear whether these non-responders have the same characteristics with those interviewed and if not, what influence on the results.

## Conclusions

This study provided no evidence of overuse of delivery services among women reimbursed by health insurance in the study areas. Furthermore, a substantial reduction of hospital costs and women's costs was found in the NCMS group, subsequently a significant alleviation on women's perceived financial affordability.

## Competing interests

The authors declare that they have no competing interests.

## Authors' contributions

SX has contributed to the preparation of the research protocol, field management and coordination, collection and analysis of data and manuscript writing. HY was responsible for design of research and sampling, and preparation of protocol, guidance on the data analysis. YS contributed to field management and coordination, preparation of the research protocol, collection and analysis of the data. SD contributed to the preparation of the research protocol, data analysis and interpretation of results. EH contributed to the preparation of the research protocol, design of the research idea, reviewing and revising the manuscript. DW contributed to the preparation of the research protocol, data analysis, interpretation of results, reviewing and revising the manuscript. QL contributed to interpretation of results, language editing and revision of the manuscript. JG contributed to the preparation of the research protocol, field management, coordination and interpretation of results. All authors read and approved the final manuscript.

## Pre-publication history

The pre-publication history for this paper can be accessed here:

http://www.biomedcentral.com/1471-2458/10/695/prepub

## Supplementary Material

Additional file 1 **Appendix Questionnaire**. The questionnaire was developed and used in the present study. Click here for file
