# Supplementary material for: Utilization of delivery care among rural women in china: does the health insurance make a difference? a cross-sectional study
Source: BMC Public Health. 2010 Nov 15;10:695. doi: 10.1186/1471-2458-10-695 (PMC3091584; doi:10.1186/1471-2458-10-695)
Supplement: Additional file 1 — Appendix Questionnaire. The questionnaire was developed and used in the present study. [file 1471-2458-10-695-S1.DOC]

**Appendix questionnaire**

Code of questionnaire _ _ _ _ _ _ _ _ _

Survey place _________province _________county _______township _______village

Name of Mother________________

Investigation date 20__ __ /__ __ /__ __ (yyyy/mm/dd)

A Background

A1 (A1a) What place your Huko was registered during your pregnancy and delivery? _________province _________county _______township

(A1b) Was your Huko (1) urban Huko (2) rural huko (3) I don’t know

A2 (A2a) Where did you live in the first three months of pregnancy?

(1) the same township where your Huko registered

(2) different township but same county__________________(A2b) (name of the township)

(3) different county

(4) I don’t know

A3 (A3a) Where did you live in the last three months of your pregnancy (26 weeks ~40 weeks of pregnancy)?

(1) the same township where your Huko registered

(2) different township but same county__________________(A3b) (name of the township)

(3) different county

(4) I don’t know

A4 (A4a) Where did you live when it was time to deliver?

(1) the same township where your Huko registered

(2) different township but same county__________________(A4b) (name of the township)

(3) different county

(4) I don’t know

A5 How old are you? ____________ (real-year-old, not nominal age)

A6 (A6a) What is your education?

(1) illiteracy or semi- illiteracy (2) primary school (3) middle school (4) high school (5) college or higher

(A6b) How many years have you studied? (If the woman did not complete a school or she does not know her exact educational level)

A7 What is your occupation?

1. Farmer (agriculture, forestry, animal husbandry and fishery)

2. city - farmer – labourer

3. rural farmer – labourer

4. urban and rural unemployed and semi-unemployed

5. industrialist without agricultural residence registration

6. Private commercial household

7. attendant in the tertiary industry

8. Governor of government agency or institution

9. Senior or secondary executive in large or medium-sized enterprise (not the owner)

10. Owner of private enterprise

11. Professional technical personnel

12. The staff of company or some kind of department

13. Student

14. Retired

A8 (A8a) Where was your husband's Huko registered during your pregnancy and delivery? _________province _________county _______township

(A8b) Was your husband's Huko

(1) urban Huko (2) rural Huko (3) I don’t know

A9 How old is the father of child? ____________(real-year-old, not nominal age)

(If information about the father cannot be provided, go to question A12)

A10 (A10a) What is the educational level of baby’s father?

(1) illiteracy or semi- illiteracy (2) primary school (3) middle school (4) high school (5) college or higher

(A10b) How many years father of the child have studied? _________ (If the man did not complete a school or she does not know his exact educational level)

A11 What is father’s occupation?

1. Farmer (agriculture, forestry, animal husbandry and fishery)

2. city - farmer – labourer

3. rural farmer – labourer

4. urban and rural unemployed and semi-unemployed

5. industrialist without agricultural residence registration

6. Private commercial household

7. attendant in the tertiary industry

8. Governor of government agency or institution

9. Senior or secondary executive in large or medium-sized enterprise (not the owner)

10. Owner of private enterprise

11. Professional technical personnel

12. The staff of company or some kind of department

13. Student

14. Retired

A12 (A12a) If you can not provide the information about the father of the child, why?

(1) father is dead (2) father is unknown (3) you are divorce (4) other reasons__________(A12b)

A13 What was the size of your family before you deliver the baby?_________ (99＝I don’t know)

A14 What was the total income of your family during your pregnancy __________________Yuan (99＝I don’t know)

A15 What was the total expenditure of your family during your pregnancy _______________Yuan （99＝I don’t know)

A16 How long does it take from your home to the village clinic using the ordinary transportation? ____________minutes

A17 How long does it take from your home to the township hospital using the ordinary transportation? ____________minutes

A18 Have you been a member of the New Cooperative Medical Scheme (NCMS) during pregnancy? (If woman does not choose (1) then skip to question B1)

(1) Yes (2) No (3) I don't know

A19 (A19a) If you have been a member of the NCMS, did you apply for maternal care reimbursement?

(1) Yes (2) No (3) I don't know

(A19b) If no, why?

(1)Illegal birth (2)birth out of wedlock (3)migrant (4)don’t know the procedure of reimbursement (5)other,___________________(A19c)

A20 Do you know whether you can apply for reimbursement for any of the services?(multi-choices)

(1) Prenatal visits (2) Hospital delivery (3) Postnatal visits （4）I don’t know

B Earlier pregnancies and Index pregnancy

B1 (B1a) Had you been pregnant before your last child?

(1)Yes (2)No

If Yes, How many pregnancies have you had ______(B1b)

If Yes, How many spontaneous abortions have you had _______(B1c)

If Yes, How many induced abortions have you had _______ (B1d)

If Yes, How many stillbirths have you had _______(B1e)

How many children do you have now?______________(B1f)

Your first child: (B1g) gender________(1=boy, 2=girl, 3=I don’t know)

(B1h) birthday_________________

Your second child: (B1g) gender________(1=boy, 2=girl, 3=I don’t know)

(B1h) birthday_________________

Your third child: (B1g) gender________(1=boy, 2=girl, 3=I don’t know)

(B1h) birthday_________________

Your forth child: (B1g) gender________(1=boy, 2=girl, 3=I don’t know)

(B1h) birthday_________________

B2 (B2a) Was your last child healthy at birth?

(1) Yes (2) No (3) I don’t know

(B2b) If not, what kind of problems did the baby have?__________________________

B3 (B3a) Is your last child healthy now?

(1)Yes (2) No (3) I don’t know

(B3b) child is sick. Why_____________________________

(B3c) child is dead. When__ __ __ __ /__ __ /__ __ (date, yyyymmdd)?

B4 (B4a) Birth weight of the baby______ Jin _____Liang. (1 Jin=1/2 kilogram, and 1 Jin=10 Liang)

(B4b) If weight is unknown how big was your baby at birth

(1) very small (2) somewhat small (3) ordinary (4) somewhat large (5) very large

B5 How many (B5a) weeks/ (B5b) months of pregnant you were when the delivery occurred? ________weeks/months.

(B5c) If the length of pregnancy is unknown was your child born

(1) too early (2) in time (3) too late

B6 (B6a) When you have been pregnant, did your husband live most of the time in the same home like you?

(1) Yes (2) No (3) Other, _______________________________ (B6b).

B7 Did you change your working activity during pregnancy?

(1) No, I worked the same as before the pregnancy until the last month of pregnancy or birth

(2) I worked less heavily than before pregnancy starting at (B7b)_____ months

(3) I stopped working completely starting at (B7c)______ months

B8 Have you had any prenatal (= antenatal) visits to a doctor or midwife during your pregnancy?

(1)Yes (2)No (Continue to the question B27)

B9 In which month of pregnancy did you have your first prenatal visit? _______months (999=I don’t remember)

B10 (B10a) How many prenatal visits have you had in public health facilities________

(B10b) in county level public health facilities_______ times

(B10c) in township level public health facilities_______ times

B11 (B11a) How many prenatal visits in some other health facilities have you had ________

(B11b) at village clinic_________ times

(B11c) at township or higher level private hospital ____________times

(B11d) at home__________ times

B12 If you had prenatal visits at township hospital who did advise you to go?

(B12a) Myself (1) yes (2) no

(B12b) My family advised me (1) yes (2) no

(B12c) Village doctors (1) yes (2) no

(B12d) Village family planning worker or women’s workers

(1) yes (2) no

(B12e) Doctor in township hospital (1) yes (2) no

(B12f) Staff in township family planning station (1) yes (2) no

(B12g) Other, who_____________________

B13 Have you had any problems during your prenatal visits?

(B13a) The visits took too much time (1) yes (2) no (3) don’t remember

(B13b) I had transportation problems (1) yes (2) no (3) don’t remember

(B13c) The visits were too expensive (1) yes (2) no (3) don’t remember

(B13d) My relatives didn’t like me to have visits (1) yes (2) no (3) don’t remember

(B13e) I had problems with childcare. (1) yes (2) no (3) don’t remember

(B13f) I had problems in organising the household (1) yes (2) no (3) don’t remember

(B13g) I had to work (1) yes (2) no (3) don’t remember

(B13h) The health service care was poor (1) yes (2) no (3) don’t remember

(B13i) The personnel were not kind (1) yes (2) no (3) don’t remember

(B13j) The visits were useless (1) yes (2) no (3) don’t remember

(B13k) Other problems ___________________________________

B14 (B14a) Did you visit county hospital during pregnancy?

(1) No

(2) Yes, on advice of township doctor/midwife

(3) Yes, by my own initiative

(4) Yes, other reasons_____________(B14b)

B15 During antenatal visits did somebody

(B15a) Ask if you had any problems

(1) Yes (2) No (3) Don’t remember

(B15b) Check term of pregnancy

(1) Yes (2) No (3) Don’t remember

(B15c) Ask about previous pregnancies and childbirths

(1) Yes (2) No (3) Don’t remember

(B15d) Advise on nutrition in pregnancy

(1) Yes (2) No (3) Don’t remember

(B15e) Advise on avoiding alcohol, smoking and hazardous substances

(1)Yes (2) No (3) Don’t remember

(B15f) Give counselling on labour (signs of start, what to do, what to expect)

(1) Yes (2) No (3) Don’t remember

(B15g) Discuss mode of delivery

(1) Yes (2) No (3) Don’t remember

(B15h) Advise in which situation seek care (such as problems and emergencies)

(1) Yes (2) No (3) Don’t remember

(B15i) Advise on follow-up visits

(1) Yes (2) No (3) Don’t remember

(B15j) Advise on assessing foetal movements

(1) Yes (2) No (3) Don’t remember

(B15k) How many times was your blood pressure measured? ________ (0=No 999=I don’t remember )

(B15l) How many times the urine test was made? __________ (0=No 999=I don’t remember )

(B15m) How many times blood test to test anaemia have you had? (need to explain anaemia) ________ (0=No 999=I don’t remember )

(B15n) How many other blood tests were made? __________ (0=No 999=I don’t remember )

(B15o) How many times abdomen investigation/palpation was made? ______

(0=No 999=I don’t remember )

(B15p) How many times foetal heart rate was assessed? __________ (0=No 999=I don’t remember )

(B15q) How many times ultrasound examination was made? __________ (0=No 999=I don’t remember )

B16 (B16a) Did you use maternity card during your pregnancy?

(1) No, I did not get a card

(2) No, I got a card but I did not use it

(3) Yes, I got a card but it was not filled in every visit

(4) Yes, I got a card and it was filled in every visit

(5) I don't know because the card was kept in the hospital

(6) Other, what___________________________________________________(B16b)

B17 (B17a) Did you get any written material related to pregnancy and child birth (booklets, leaflets etc) during your visits?

(1) Yes (2) No (3) I don’t remember

(B17b) If no, would you like to get it?

(1) Yes (2) No (3) I don’t know

B18 What was the quality of prenatal visits? (1 = Very good, 2 = Good, 3 = Neither good, nor bad, 4 = Bad, 5 = Very bad, 9 = I did not use)

at township hospital 1 2 3 4 5 9 (B18a)

at MCH station or at county hospital 1 2 3 4 5 9 (B18b)

B19 Would you recommend the township level health facility for prenatal visits?

(1)Yes (2) No (3) I don’t know (4) No use of care

B20 Would you recommend the county level health facility for prenatal visits?

(1)Yes (2) No (3) I don’t know (4) No use of care

B21 How much did your prenatal care cost totally?____________ Yuan

(90000=I don’t know, 99999=I don’t remember)

B22 (B22a) Did you get reimbursement from CHIMACA project?

(1) Yes (2) no (3) I don't know (4) I don't remember (B22b) if not ,why_____________

(B22c) If yes, how much?_______ Yuan (90000=I don’t know, 99999=I don’t remember)

For what type of care did you get reimbursement?

(B22d) prenatal care (1)yes (2)no

(B22e) hospital delivery (1)yes (2)no

(B22f) postnatal care (1)yes (2)no

(B22g) I don’t know (1)yes (2)no

B23 Did CMS cover your prenatal care costs? _________yuan (90000=I don’t know, 99999=I don’t remember)

B24 Did another insurance or your employer cover your prenatal care costs?

__________yuan (90000=I don’t know, 99999=I don’t remember)

B25 (B25a) How much did you pay by yourself for prenatal care?

(1) Nothing (2) _______(B25b), Yuan as a package (3) _______(B25c), Yuan, for different things (4) I don’t know

B26 If you or your family paid prenatal care services by yourselves (partly or totally), do you think the price was

(1) Far too high (2) Too high (3) Reasonable (4) Low (5) I don’t know

B27 Why did not you use the offered prenatal care service?

(B27a) I thought it was not necessary (1) yes (2) no

(B27b) I had no time (1) yes (2) no

(B27c) I had not enough money (1) yes (2) no

(B27d) I had a transportation problem (1) yes (2) no

(B27e) I was afraid of going to the higher level hospital (1) yes (2) no

(B27f) Other reason __________________________________________

B28 Did you (or your family members) search for any information related to pregnancy and child birth from the Internet?

(1) Yes (2) No (3) I don’t know

C Delivery

C1 (C1a) Where did you give birth?

(1)County or higher level hospital or maternal and child care institute, name_____________(C1b)

(2) Township hospital, name ________________(C1b)

(3) Village health clinic

(4) Family planning station

(5) At home (skip to C2)

(7) Elsewhere ________________(C1b)

C2 If you delivered at home indicate reasons

(C2a) I thought it was not necessary to go to hospital

(C2b) The delivery was quick and I had no time to go elsewhere

(C2c) I had not enough money

(C2d) The hospital was far away or it was difficult to reach

(C2e) The treatment in hospital is known to be poor

(C2f) Other ____________________________________________

C3 (C3a) Who assisted the delivery? (women delivered at home skip to D1)

(1) Midwife (2) Doctor (3) FP worker or village worker

(4) Family member (5) Someone else _____________________ (C3b) (6) Nobody

C4 (C4a) How did the birth take place (if no C-section, skip to the question C6)?

(1)Normal vaginal birth (2) Birth assisted by instrument (3) Assisted breech birth (4) Caesarean section

If birth was given by C-section what was the reason?

(C4b) "Emergency" (child's or my own condition were in danger) (1) yes (2) no

(C4c) My doctor / midwife recommended it for other than emergency reason. (1) yes (2) no

(C4d) I wanted to have it (1) yes (2) no

(C4e) Some of my family members advised me to have it (1) yes (2) no

(C4f) Other reason _______________________________________

(C4g) I don't know (1) yes (2) no

(C4h) What was the reason for your own decision to have C-section? (Choose 1~5 choices as women like)

(1) I thought it is better for the child to be born by c-section

(2) I thought it is better for the mother to give birth by c-section

(3) I was afraid of pain

(4) I was afraid of my baby's health

(5) C-section made it possible to fix the date of birth

(6) TV, radio or journals influenced my decision

(7) My friend(s) or neighbours have influenced my decision

(8) My relative(s) has (have) influenced my decision

(9) I had bad experiences from previous deliveries

(10) My relatives or friends have had bad experiences of normal delivery

(11) My relatives or friends have had good experiences of c-sections

(12) Other _____________________________________________ (C4i)

C5 (C5a) When was the decision for C-section made?

(1) In which week of pregnancy _________ (C5b) gestational weeks

(2) Just before the labour (3) During the labour (4) I don’t know

C6 (C6a) Did you get any pain relief (all kinds of methods – not only drugs) during your delivery?

(1) Yes, what kind of pain relief______________________ (C6b) (2) No

(3) I don’t remember (4) I don’t know

C7 What factors influenced your selection of the hospital

(C7a) On advise of my doctor/midwife (1) yes (2) no

(C7b) It was the closest hospital (1) yes (2) no

(C7c) It was convenient to go there (1) yes (2 )no

(C7d) I knew the doctor in the hospital (1) yes (2) no

(C7e) The care was better than in other hospitals (1) yes (2) no

(C7f) It was cheaper than in other hospitals (1) yes (2) no

(C7g) It was safer than other hospitals (1) yes (2) no

(C7h) Other reasons,_________________________________________

C8 (C8a) Why did you deliver at county hospital?

(1) Recommendation by a township doctor/midwife during prenatal visits

(2) I was transferred there during delivery (because of problems)

(3) My own or my family members decision

(4) Other reasons ________________________________________ (C8b)

C9 (C9a) Did the doctor recommend you to stay in hospital before delivery?

(1) Yes (2) No

(C9b) How many nights did the doctor recommend you to stay in hospital before delivery? ____ nights

(C9c) How many nights did you stay in the hospital before delivery? ________nights

(C9d) Why did you go to hospital later than doctor's recommendation?

(1) I had not enough money (2) It was not necessary (3) I was too busy

(3) Family advised me to (4) Relatives or friends suggested (5) other reason (C9e)____________

C10 (C10a) Did the doctor recommend you to stay in hospital after delivery? (1) Yes (2) No

(C10b) How many nights were recommended to stay in hospital after delivery? ____ nights

(C10c) How many nights did you actually stay in the hospital after delivery? ________nights

(C10d) If you left hospital earlier than the time advised by your doctor after you had delivered, the most important reason was:

(1) I had not enough money (2) It was not necessary (3) I was too busy

(3) Family advised me to (4) Relatives or friends suggested (5)other reason (C10e)___________

C11 The next question related to satisfaction or your experiences during delivery care

(1 = Very good, 2 = Good, 3 = Neither good, nor bad, 4 = Bad, 5 = Very bad, 9 = I did not use)

Professional skills (how experienced doctors and midwifes were) during the labour and your stay in hospital were

at township level health facility 1 2 3 4 5 9 (C11a)

at county level health facility 1 2 3 4 5 9 (C11b)

hospital environment of the delivery hospital was

at township level health facility 1 2 3 4 5 9 (C11c)

at county level health facility 1 2 3 4 5 9 (C11d)

Doctor/ midwife’s attitude to you in the delivery hospital was

at township level health facility 1 2 3 4 5 9 (C11e)

at county level health facility 1 2 3 4 5 9 (C11f)

C12 Would you recommend the hospital you delivered?

(1) Yes (2) No (3) I don’t know (4) I didn’t use

C13 How much did your delivery care cost totally?____________ Yuan

(90000=I don’t know, 99999=I don’t remember)

C14 Did CMS cover your delivery care costs? _________yuan (90000=I don’t know, 99999=I don’t remember)

C15 Did other insurance or your employer cover your delivery care costs?

__________ yuan (90000=I don’t know, 99999=I don’t remember)

C16 (C16a) How much did you pay by yourself for delivery care?

(1) Nothing (2) _______ (C16b), Yuan as a package (3) _______(C16c), Yuan, for different things, (4) I don’t know

C17 If you or your family paid delivery care services by yourself (partly or totally), do you think the price was

(1) Far too high (2) Too high (3) Reasonable (4) Low (5) I don’t know

D Postnatal care

D1 (D1a) During 42days after delivery how many postnatal visits did you have at (do not calculate telephone calls)?____________ (I don’t remember 99 )

(D1b) at home__________ times

(D1c) In county level health facility ______times

(D1d) In township level health facility _____times

(D1e) At village clinic_____times

(D1f) Other health facility ________________________, ________(D1g) times

D2 Did health care worker (at least once)

(D2a) Ask about your feelings (1) Yes (2) No (3) Don’t remember

(D2b) Ask you about problems related to you or your baby

(1) Yes (2) No (3) Don’t remember

(D2c) Check your blood pressure

(1) Yes (2) No (3) Don’t remember

(D2d) Check your temperature

(1) Yes (2) No (3) Don’t remember

(D2e) Palpate abdomen to check uterus involution

(1) Yes (2) No (3) Don’t remember

(D2f) Check the amount, colour and smell of lochia (bleeding)

(1) Yes (2) No (3) Don’t remember

(D2g) Examine breasts and ask about breastfeeding

(1) Yes (2) No (3) Don’t remember

(D2h) Advice how to seek for health care

(1) Yes (2) No (3) Don’t remember

(D2i) Advice on hygiene in postpartum period

(1) Yes (2) No (3) Don’t remember

(D2j) Advice on nutrition

(1) Yes (2) No (3) Don’t remember

(D2k) Advice on FP and sexual relations

(1) Yes (2) No (3) Don’t remember

(D2l) Advice on breastfeeding

(1) Yes (2) No (3) Don’t remember

(D2m) Examine the baby

(1) Yes (2) No (3) Don’t remember

(D2n) Weigh the baby

(1) Yes (2) No (3) Don’t remember

(D2o) Advice on baby care

(1) Yes (2) No (3) Don’t remember

D3 (D3a) If you received information from health care workers only by phones, how many calls did you get?_________ (I don’t remember 99 ) Who did call you? ________________

D4 (D4a) When did you breast feed your baby for the first time?

(1) Within half an hour after the delivery (2) Within 24 hours after the delivery

(3) Later than 24 hours after the delivery (4) Never Why?______________________ (D4b)

D5 How long did you only breast feed your baby? _____________months

D6 (D6a) When did you start to give milk substitute to your baby at the first time (excluding the possible substitute given just after the birth before the breastfeeding has really started)?

(1) I started _________(D6b) months

(2) I have not started yet, but I plan to give at ______(D6c) months

(3) I have not planned to give milk substitute at all

(4) I don't know

D7 (D7a) Did your baby get vaccination? (check the vaccination card)

(1)yes,_____(D7b)times and _____ (D7c) types (2) no (3)I don’t know

D8 Postnatal care during the visits was (1 = Very good, 2 = Good, 3 = Neither good, nor bad, 4 = Bad, 5 = Very bad, 9 = I did not use)

at home 1 2 3 4 5 9 (D8a)

at township hospital 1 2 3 4 5 9 (D8b)

at MCH station or at county hospital 1 2 3 4 5 9 (D8c)

D9 How much did your postnatal care cost totally?____________ Yuan

(90000=I don’t know, 99999=I don’t remember)

D10 Did CMS cover your postnatal care costs?_________yuan (90000=I don’t know, 99999=I don’t remember)

D11 Did another insurance or your employer cover your postnatal visits costs?

_________yuan (90000=I don’t know, 99999=I don’t remember)

D12 (D12a) How much did you pay by yourself for postnatal care?

(1) Nothing (2) _______(D12b), Yuan as a package (3) _______(D12c), Yuan, for different things. (4) I don’t know

D13 If you or your family paid postnatal care services by yourself (partly or totally), do you think the price was

(1) Far too high (2) Too high (3) Reasonable (4) Low (5) I don’t know

E The degree of cooperating of the responder

(1) very good (2) good (3) common (4) bad (5) very bad

checking

Time of the first checking Signature：__________

Time of the second checking Signature：__________

Time of the third checking Signature：__________
